# The Use of Botulinum Toxin for the Treatment of Chronic Joint Pain: Clinical and Experimental Evidence

**DOI:** 10.3390/toxins12050314

**Published:** 2020-05-10

**Authors:** Nicole Blanshan, Hollis Krug

**Affiliations:** 1 Minneapolis VA Health Care System, Minneapolis, MN 55455, USA; nicole.blanshan@va.gov; 2 Department of Medicine, University of Minnesota, Minneapolis, MN 55455, USA

**Keywords:** pain, botulinum toxin, arthritis, osteoarthritis, intra-articular

## Abstract

Chronic osteoarthritis pain is an increasing worldwide problem. Treatment for osteoarthritis pain is generally inadequate or fraught with potential toxicities. Botulinum toxins (BoNTs) are potent inhibitors of neuropeptide release. Paralytic toxicity is due to inhibition at the neuromuscular junction, and this effect has been utilized for treatments of painful dystonias. Pain relief following BoNT muscle injection has been noted to be more significant than muscle weakness and hypothesized to occur because of the inhibition of peripheral neuropeptide release and reduction of peripheral sensitization. Because of this observation, BoNT has been studied as an intra-articular (IA) analgesic for chronic joint pain. In clinical trials, BoNT appears to be effective for nociceptive joint pain. No toxicity has been reported. In preclinical models of joint pain, BoNT is similarly effective. Examination of the dorsal root ganglion (DRG) and the central nervous system has shown that catalytically active BoNT is retrogradely transported by neurons and then transcytosed to afferent synapses in the brain. This suggests that pain relief may also be due to the central effects of the drug. In summary, BoNT appears to be safe and effective for the treatment of chronic joint pain. The long-term effects of IA BoNT are still being determined.

## 1. Introduction

Osteoarthritis (OA) is a worldwide problem. It is the most common cause of chronic joint pain and is increasing in prevalence. The prevalence of OA increases with age, and as the population ages, the number of people with OA is expected to increase. In the US alone, 27 million adults are affected by this disease, and by the year 2030, this number is expected to reach 67 million [1,2]. Disability from osteoarthritis varies, but the pain is the most disabling symptom [3,4]. Arthritis is a leading cause of work disability among adults in the US [5]. Because there are no treatments for OA that modify disease, treatment goals must be focused on the relief of symptoms and minimizing disability. The management of chronic pain from OA is a challenge. Nonpharmacologic methods, such as weight loss, joint protection, and acupuncture, are generally insufficient to provide enough joint pain relief to reduce disability. Pharmacologic therapies can include systemic, topical, and intra-articular medications [6]. These therapies can be problematic with intolerable side effects, insufficient pain relief, and dangerous drug interactions, increasing the risk/benefit ratio [7]. Opioids are controversial in the treatment of chronic musculoskeletal pain. They have not been shown to be more effective than other forms of pain control, and the rising opioid epidemic and risk of overdose, make this option increasingly unattractive [8,9]. Treatment of OA pain in the elderly is particularly problematic. They are more likely to have advanced OA and are more intolerant to usual medical therapies than younger individuals [10]. Surgical therapies, such as total joint replacement, have an established place in the treatment of end-stage OA but are not appropriate for less severe disease. Knee joint lavage is not more effective for OA pain than a placebo [11,12]. Intra-articular therapies, such as corticosteroids and hyaluronic acid preparations, have not been clearly demonstrated to be effective. Because of this, the Academy of Orthopedic Surgeons recommends against viscosupplementation and believes that the evidence for corticosteroid injections for OA is inconclusive [13].

Because current therapies are inadequate, new therapies designed to directly reduce nociception have begun to be developed. These include systemic therapy with monoclonal antibodies directed against nerve growth factor (NGF), intra-articular vanilloids, such as highly purified synthetic trans-capsaicin (CNTX-4975), and intra-articular onabotulinumtoxinA [14,15,16]. This report reviewed the current data, supporting the use of onabotulinumtoxinA for chronic joint pain, and presented some pre-clinical data to support the use of this toxin in humans.

### 1.1. Neurobiology of Chronic Pain

Pain is the result of noxious signal transmission via peripheral nerves to the spinal cord, and finally the brain, where it is perceived as pain. Most pain is the result of some sort of injury, at least initially. Pain signals the organism to withdraw or avoid the stimulus producing the pain in order to avoid damage. Chronic pain occurs as a result of alterations of this system so that there is no longer a direct relationship between pain perception and the noxious stimulus. The nervous system has a certain amount of plasticity that allows peripheral nerves to become more sensitive to stimuli or produces spinal cord neurons that demonstrate increased excitability. There are also descending inhibitory pathways originating in the midbrain and brainstem that reduce pain sensitivity. These pathways can be altered by projections from the spinal cord. The sum of these changes results in an altered perception of any stimulus and leads to persistent pain. These changes appear to be reversible and can be modified with pharmacologic therapies [17].

Increased peripheral sensitivity to nociception can be the result of inflammation or nerve injury, which sensitizes nociceptive receptors resulting in increased intracellular calcium, activated tyrosine kinases, and intracellular protein kinase C. This increased activation induces phosphorylation of sodium channels on sensory neurons and receptors for transient receptor potential vanilloid 1 (TRPV1), causing a reduced pain threshold. Peripheral neurons can release chemical stimulants to the nervous system, such as substance P (SP) and calcitonin gene-related peptide (CGRP). These neuropeptides amplify the inflammatory response by acting on blood vessels and inflammatory cells, causing vasodilation, edema, and increased local inflammation. This inflammation only adds to the peripheral sensitization [18].

### 1.2. Botulinum Toxin

Botulinum toxins are the most lethal toxic proteins known. All botulinum toxins are products of the bacterial genus *Clostridium.* There are eight serotypes. Seven of these, types A–G, have varying durations of action and enzymatic targets. All have been fully characterized. The genetic sequence of the recently described eighth serotype, H, has been withheld due to public safety concerns. It is considered the deadliest substance in the world [19]. Poisoning with botulinum toxin can occur as the result of an infection in a wound; preformed toxin can be ingested in contaminated food, or in infants, it can occur as the result of colonization of the infant’s gut by bacteria that produce the toxins in situ. Botulism, the state of intoxication with botulinum toxin, results in paralysis of the muscles of the skeletal system, including muscles affecting the respiratory system, and visceral muscles, including the heart. In addition, dysfunction of the autonomic nervous system results in reduced salivation and tear formation, nausea, vomiting, and abdominal pain; in infants, weakness, hypotonia, feeble crying, ptosis, and apnea. Although most individuals survive botulism, recovery is slow and may require mechanical ventilation [20].

### 1.3. Botulinum Toxin Mechanism of Action

Botulinum toxins function by cleaving soluble N-ethylmaleimide-sensitive factor (NSF) attachment protein receptor (SNARE). SNARE proteins function to bring the synaptic vesicle membrane, and the terminal plasma membrane of the peripheral nerve, together to allow fusion of the two membranes and release of neurotransmitters, such as acetylcholine (Ach). When the SNARE proteins are cleaved, this fusion cannot happen, and the neurotransmitter release is impaired. This inability to release substances, such as Ach, produces the paralytic activity of the toxins [20]. Botulinum toxins consist of a light chain and a heavy chain linked through a disulfide bridge [21]. The heavy chain of Botulinum toxin A binds to nerve endings at the C-terminal half of the heavy chain, while the light chain enters the cytosol by vesicle endocytosis and cleaves soluble NSF attachment protein-25 (SNAP-25), preventing neurotransmitter release [22]. Different serotypes have slightly different potencies and speed of onset depending on different heavy and light chain mechanistic differences. Chimeras of the heavy and light chains of different serotypes can impart these different characteristics to the combined protein [23].

Because of their paralytic effects, botulinum toxins A and B have been used to treat painful muscle dystonias, such as torticollis. Initially, it was thought that the paralytic effect on the muscles was responsible for the pain relief that these treatments afforded, but it was observed that the pain relief preceded the muscle weakness that was the result of the toxin treatments. This observation led to early pilot studies of intra-articular botulinum toxin (Type A) (BoNT/A) for end-stage osteoarthritis pain [24]. Other studies have confirmed that not only does botulinum toxin inhibit the release of Ach, but it also inhibits nociceptive neurotransmitters, such as substance P (SP), calcitonin gene-related protein (CGRP), and excitatory neurotransmitters, such as glutamate [25,26,27]. Translocation of neurotransmitter receptors, such as the N-methyl-D-aspartate (NMDA) receptor and transient receptor potential vanilloid 11 (TRPV1), to the neural cell membrane is also inhibited by botulinum toxin [28,29]. These receptors are important in many functions related to nociception. The NMDA receptor binds glutamate, an excitatory neurotransmitter, and TRPV1 signals pain due to heat, acid, and vanilloids, such as capsaicin.

## 2. Materials and Methods

A literature review for this paper was performed using PubMed with the search terms “botulinum toxin, joint, and pain”. Criteria for inclusion for both animal and human studies were that the toxin was delivered by intra-articular injection. Studies of botulinum toxin for joint pain that used only intramuscular injection were not included. In addition to the above search terms, meta-analyses that were identified with these search terms were reviewed for additional appropriate studies to include in this review. This review was not a meta-analysis. Quality of the few studies that were identified was not criteria for inclusion but was discussed.

## 3. Results

### 3.1. Clinical Use of Botulinum Toxin for Arthritis Pain

Only a limited number of clinical trials using botulinum toxin for articular pain have been done in humans.These were initiated before studies were done in preclinical models due to the observation that the use of botulinum toxin for muscular dystonias relieved pain out of proportion to the production of muscle weakness. Meta-analyses and systematic reviews have been published, summarizing these results [24,30,31,32,33]. A meta-analysis of six randomized controlled trials using IA BoNT for osteoarticular pain, found a statistically significant, short-term one point decrease of a numerical rating scale (NRS) pain score, and a decrease at 6 mo that was not statistically significant [34]. An earlier meta-analysis, which included some of the same studies, found similar results [33]. Doses of onabotulinumtoxinA (BOTOX) administered per joint were between 100 and 200 IU, and abobotulinum toxin A (Dysport) doses were 200–500 IU. The dose of rimabotulinumtoxinB (Myobloc) was 2500 IU. These studies used varying controls, ranging from triamcinolone to saline to unspecified placebo. Some studies used botulinum toxin diluted with lidocaine and compared to saline with lidocaine. One study of 75 patients compared intra-articular (IA) BoNT/A to IA injection of 2 mL sodium hyaluronate in symptomatic OA ankles and found no difference in effectiveness between the two treatments [35]. It is unclear whether this comparison is appropriate since the American Academy of Orthopedic Surgeons evidence-based guidelines for the treatment of OA of the knee state that viscosupplementation cannot be recommended due to lack of evidence for effectiveness [36]. In the largest double-blind, randomized, placebo-controlled study of botulinum toxin for articular pain to date, 121 patients with knee pain were randomized to receive IA onabotulinumtoxinA or placebo [16]. Outcome measures included pain biomarkers to measure both localized and widespread sensitization, and wind-up pain, which measured central sensitization. Clinical outcomes included the Western Ontario and McMaster Universities Osteoarthritis Index (WOMAC), average daily pain, patient global impression of change, and rescue medication. Patients were grouped into specific subgroups using the pain DETECT questionnaire. Subgroups included nociceptive pain (56.2%), neuropathic pain (11.6%), and mixed/uncertain (32.2%). Although all the subgroups were identical at baseline with respect to OA characteristics and pain testing, the nociceptive group showed significant improvement after IA onabotulinumtoxinA at week 8 for WOMAC outcomes, for pain at weeks 9 and 10, and for a global impression of change at week 12. There was also a significant reduction in rescue medication at weeks 9 and 10. The neuropathic pain group did not demonstrate the same improvement. The authors concluded that IA onabotulinumtoxinA given to patients with nociceptive knee OA pain reduced pain sensitization and improved pain and function [16]. There have not been significant safety issues in any of the studies of IA BoNT. Weakness was a concern due to the known paralytic activity of this toxin, but no significant weakness was found in any clinical trial [16,24,31]. In fact, since pain relief may last up to 6 months, less frequent injections may be required compared to other IA therapies, resulting in reduced risk of infections or other complications. More recently, a trial of abobotulinumtoxinA injected into the hip adductor muscles of patients with hip OA was performed. Injection of 400 units abobotulinum toxin A improved the primary outcomes of visual analog scale (VAS) pain scores and Harris hip scores but did not change secondary outcomes of muscle strength or Short Form (SF)-36 scores [37]. The choice of placebo comparison needs to be considered when evaluating these treatments. Saline has been suggested to have efficacy on its own, so when comparing results to saline control, a complete lack of efficacy may be incorrectly concluded [38]. In addition, IA placebo injections have been shown to have a medium effect size on efficacy, further confusing the picture [39]. Table 1 summarizes the results of the studies of IA BoNT for joint pain.

### 3.2. Preclinical Models for the Study of Botulinum Toxin for Arthritis Pain

Since BoNT was already approved for clinical use, the first studies of BoNT for joint pain were conducted in humans. These studies led to many questions about the mechanism of action of neurotoxins for the treatment of joint pain and about the potential toxic adverse effects. Naturally, preclinical models were used to try to answer these questions. Studies of IA BoNT for joint pain have been conducted in mice, rats, and dogs (Table 2). It appears to be effective for chronic joint pain but not for acute pain in mice, supporting the hypothesis that the toxin inhibits neuropeptide release in the periphery and, consequently, inhibits peripheral sensitization [50,51]. A single study evaluated the effectiveness of rimabotulinumtoxinB (BoNT/B) for OA pain in mice and found reduced spontaneous, as well as evoked pain behavior [52]. In a rat model of adjuvant-induced arthritis in the tibial-tarsal joint, IA BoNT/A improved mechanical and thermal hyperalgesia and reduced the number of dorsal root ganglion (DRG) cells doubly positive for TRPV1 and CGRP, indicating reduced TRPV1 expression in the DRG. This occurred without reducing TRPV1 transcription, confirming the importance of SNARE proteins in the translocation of this receptor to the cell membrane. Retrograde axonal transport of BoNT/A was confirmed by demonstrating the presence of the BoNT/A cleavage product, cl-SNAP-25, co-localized with TRPV1 in the ipsilateral DRG [53]. Another rat model of inflammatory arthritis in the temporomandibular (TMJ) joint was treated with BoNT/A. This treatment reduced pain behaviors that resulted from the injection of low dose formalin into the inflamed joints. In this model, the trigeminal ganglion of BoNT/A-treated animals released less SP and CGRP than saline-treated animals. Glutamate and its receptors, AMPA (α-amino-3-hydroxyl-5-methyl-4-isoxazole-propionate) and NMDA (N-methyl-D-aspartate), were unchanged with treatment. The BoNT/A treatment reduced the release of interleukin 1 beta (IL-1β) but had no effect on the release of tumor necrosis factor alpha (TNFα) in these inflamed joints [54]. In dogs, the osteoarthritic synovial fluid contained a higher level of prostaglandin E2 (PGE2) than normal joints, and levels negatively correlated with peak vertical force, vertical impulse, and positively with tenderness. Interestingly, IA BoNT had no effect on the levels of PGE2 or SP in SF [55]. Another study of IA BoNT for pain management in dogs with OA did not find better pain control compared to IA saline. The outcome measures in this study were less objective than the Heikkila study, with clinical evaluation by a veterinarian who completed a clinical score based on function and owners who completed two descriptive pain questionnaires. Rescue analgesia was allowed but not required by any subject. Both groups improved after treatment, but there was no statistical difference [56].

### 3.3. The Effect of Botulinum Toxin on the Central Nervous System

Our understanding of the analgesic effects of botulinum toxin in the musculoskeletal system is incomplete. Initial theories suggested that the effect was entirely due to action on the peripheral nervous system and the reduction of local sensitization. However, clinical studies of BoNT for dystonia have suggested that the muscle-weakening effects may also be the consequence of BoNT’s action at the spinal and supraspinal levels [60]. Pain responses are out of proportion and discordant with relevant muscle weakness. The benefit may occur even with very small doses, and effects may last longer than anticipated after only one or a few doses [61]. Neurophysiologic studies of patients with lower limb spasticity demonstrated an effect of BoNT on spinal synaptic transmission, thought to be due to effects on inhibitory interneurons in the spinal cord through retrograde transport [62].

Botulinum toxins vary with respect to their physical and chemical characteristics and in their systemic or contralateral effects. The functional toxin is a protein complex, which may have different molecular weights depending on whether the toxin is bound to hemagglutinin, and if so, how many subcomponents of hemagglutinin are bound. Similarly, different subtypes of the botulinum organism produce toxins with slightly different amino acid sequences. Type A *Clostridium botulinum* has five subtypes. The largest, type A1 toxin, has been compared in rats and in primary spinal cord cells to the smallest, type A2 toxin. The smaller A2 toxin enters the cells more quickly and thus provides a more potent blockade of the neuromuscular junction. However, it is transported less effectivelyin the rat foreleg muscles compared to the larger A1 toxin. There are also differences in the type of inhibition, depending on the type of synaptic transmission. The smaller A2 is carried mainly to contralateral muscles via the blood, whereas the larger A1 toxin travels via both blood and spinal neurons [63].

Both BoNT/A and BoNT/B inhibit neurotransmitter release when administered in the periphery and are taken up by the peripheral terminal and transported to the DRG [27,64]. BoNT/A has been found to reduce protein levels of TRPV1 in the DRG of rats with adjuvant arthritis without reducing mRNA expression, suggesting the inhibition of plasma membrane trafficking of the receptor [53]. BoNT/A may produce analgesia through interaction with the endogenous opioid system since naltrexone abolishes the antinociceptive and anti-inflammatory effects of BoNT/A in rats [65].

In preclinical models, not only has BoNT been shown to be transmitted in a retrograde fashion to the DRG, but cleaved SNAP-25 has also been found in the contralateral cortex in mice when injected into the ipsilateral hippocampus. This suggests that catalytically active BoNT is retrogradely transported by neurons and then transcytosed to afferent synapses. Interestingly, this transport only occurs with botulinum A and not with botulinum E, which has a much shorter duration of action in the brain. This translocation of cleaved SNAP-25 has resulted in changes in function in the untreated hemisphere [66]. This group later showed that it was indeed BoNT/A that was being transcytosed and not merely the SNAP-25 cleavage product. Other studies have confirmed that BoNT/A administered unilaterally reversed hyperalgesia bilaterally [67]. These observations suggest that the effect of BoNT treatment may be explained by an indirect central effect mediated by changes to sensory afferents. This may lead to modulation or reorganization of the brain, providing the observed additional therapeutic effects that cannot be explained solely by the peripheral actions. In fact, BoNT/A modulates the proliferation of Schwann cells and inhibits the acetylcholine release from Schwann cells, suggesting a new biological combinatorial effect of the toxin and influence on regenerative processes [68].

A botulinum toxin A-based molecule—BiTox—has been synthesized that is reported to retain neuronal silencing capacity without causing paralysis. This molecule reduces plasma extravasation and inflammatory edema but is not transported to the DRG or dorsal horn, does not inhibit pain behaviors in response to formalin or capsaicin, and does not inhibit formalin-induced c-Fos expression in the dorsal horn. It has been found to strongly reduce A-nociceptor-mediated secondary mechanical hyperalgesia due to CFA joint inflammation or capsaicin injection and decreased hypersensitivity from nerve injury. The authors concluded that this botulinum toxin-based molecule could reduce the local release of neuromodulators from C fibers without impairing C nociceptive signaling function [59].

## 4. Discussion

Neurotoxins are being increasingly proposed and studied for use for chronic joint pain. Clinical evidence suggests that IA BoNT is effective for musculoskeletal pain, but these effects are incomplete, and efficacy appears to be limited to individuals with nociceptive pain. The strength of the effect seems to be related to the comparator, but some placebo treatments, such as saline, may have efficacy of their own. Clinical trials have generally been small, with varying comparator substances, different outcome measures, and in different painful joints. Most, but not all, have been studied in osteoarthritis. Few have included inflammatory joint disease. For these reasons, the results are difficult to interpret, and comparisons cannot effectively be made between the studies. In addition, the magnitude of the placebo effect of intra-articular injection cannot be ignored. The two largest studies, to date, both used saline as a comparator but came to opposite conclusions [16,42]. This may have been due to incomplete characterization of the pain since the Arendt-Nielsen study differentiated neuropathic from nociceptive pain. This stratification also reduced the number of relevant study participants.

Likewise, analgesic studies of IA BoNT in preclinical models are diverse and varied with respect to the type of arthritis model, joint involved, species, pain outcome measures, dose of botulinum toxin, the serotype of toxin, and comparator substance. Since osteoarthritis is the main driver of chronic joint pain in humans, animal models of OA pain would be ideal for studies of the efficacy of BoNT for joint pain. Unfortunately, OA pain is difficult to measure in preclinical models. Mice are particularly difficult. Dogs may be a better choice for pain outcome measurement, but studies would be costlier, and histochemical and neurologic studies more difficult. Rat models of OA exist, but there is much less information about the pain response in rats with this disease. Inflammatory arthritis models in mice seem to produce more pain that is easier to quantify, but this model may not be relevant for treating OA in humans. Studies in animal models may not be able to clarify the effect of the placebo response seen in humans.

The effects on the central nervous system of IA BoNT have not been completely determined. It seems there may be central effects contributing to analgesia. To date, no significant toxicity has been reported for BoNT given intra-articularly, and administration is simple. Studies are ongoing in preclinical models, as well as in humans, to determine the long-term effects of IA BoNT on the central nervous system.

In summary, the use of BoNT for the treatment of chronic joint pain appears to be safe, available, and clinically feasible. It may a reasonable alternative for individuals with no other therapeutic options. Much more information is needed from clinical and preclinical studies to determine which patients will benefit most from this therapy.

## Figures and Tables

**Table 1:** Human trials of IA botulinum toxin (BoNT) for the treatment of arthritis pain.

Author/Year	Comparator	Number of Participants	Study Design/Primary Outcome	Joint Treated	Results	Reference
Najafi/2019 Uncontrolled, open-label	none	24	100 IU BoNT/A Knee pain at 4 weeks	Knee OA	Significantly reduced pain from baseline	[40]
Mendes/2019 Double-blind RCT	Triamcinolone (TC) and Saline	105	100 IU BoNT/A, 40 mg TC, saline 1:1:1 Pain VAS 4, 8, 12 weeks	Knee OA KL II and III	VAS pain NS at 12 weeks b/t 3 groups TC superior at 4 weeks	[41]
McAlindon/2018 RCT	Saline	176	400 IU/200 IU IA BoNT/A/saline 1:1:2 Pain	Knee OA	No significant difference in pain	[42]
Bao/2018 RCT	Saline and HA	60	100 IU BoNT/A, saline, HA 1:1:1 with exercise in all groups WOMAC and VAS pain	Knee OA KL II and III (one IV)	BoNT/A superior to HA and saline in pain, function	[43]
Arendt-Nielsen/2017 Double-blind RCT	Saline	121	200 IU BoNT/A, saline Pressure pain Thresholds, WOMAC, average daily pain and worst pain, Patient global impression of change at 16 weeks	Knee OA KL I, II, and III	Reduced pain sensitization, improved WOMAC, Patientglobal impression of change, and pain in the nociceptive group only (68)	[16]
Hsieh /2016 RCT	Education	46	100 IU IA BoNT/A Pain 1 week and 6 months	Knee OA	Significant improvement 1 week and 6 months	[44]
Sun/2014 RCT	Hyaluronate	75	100 IU BoNT/A, 2 mL Hyaluronate Pain VAS, ankle OA scale at 6 months	Ankle OA	Significant improvement up to 6 months in both groups	[35]
Joo/2013 RCT	Triamcinolone acetate (TA)	28	200 IU BoNT/A vs. TA 20 mg Pain NRS at 4 and 8 weeks	Shoulder adhesive capsulitis	No difference between the groups	[45]
Singh/2010 RCT	Saline	49	100 IU BoNT/A vs. Saline VAS, McGill, WOMAC at 2, 3, and 4 months	Painful TKA	Significantly increased % pts with clinically significant reduction in pain VAS	[46]
Boon/2010 Double-blind RCT	40 mg Methylprednisolone	60	100 IU BoNT/A, 200 IU BoNT/A, 1:1:1 Pain VAS at 8 weeks	Knee OA KL II and III	Pain improved at 8 weeks in 100 IU group	[47]
Chou/ 2010 Non- randomized, open-label	None	24	100 IU BoNT/A X2 T0 and 3 months WOMAC pain and stiffness	KL III and IV Knee OA	WOMAC pain and stiffness improved at 3 months	[48]
Singh/2009 RCT	Saline + Lidocaine	43	100 IU boNT/A + lidocaine 1:1 VAS pain, SPADI disability	Refractory shoulder pain	Significant pain relief and improved quality of life in BoNT/A group at 1 month	[49]

RCT—randomized controlled trial, HA—hyaluronan, OA—osteoarthritis, TKA—total knee arthroplasty, VAS—visual analog scale, NS—not significant, IA—intra-articular, WOMAC—Western Ontario McMaster Universities Arthritis Index, McGill—McGill Pain Questionnaire, SPADI—shoulder pain and disability index.

**Table 2:** Preclinical studies of botulinum as analgesics for arthritis pain.

Arthritis Model	Experiment	Results	Reference
Murine CFA, COL	IA BoNT/A vs. sham	Reduced spontaneous and evoked pain behaviors in CFA arthritis, reduced spontaneous pain behavior in COL arthritis	[50,51]
Murine COL	IA BoNT/B vs. saline or sham injection	Improved visual gait analysis, improved joint tenderness	[52]
Dog, hip OA	IA BoNT/A vs. saline	BoNT did not provide better pain relief than a control	[56]
Dog, OA, multiple joints	IA BoNT/A vs. placebo Outcome measured after 12 weeks	Improved peak vertical force, improved Helsinki chronic pain index, no effect on synovial fluid SP or PGE2 levels	[55,57]
Healthy Beagles (safety study)	IA BoNT vs. placebo	No adverse clinical, cytological, or histopathological effects. Some EMG evidence for spread outside the joints to muscle	[58]
Rat BSA TMJ inflammatory arthritis	IA BoNT/A followed by pain induction with formalin injection	Significantly reduced pain behaviors, reduced SP and CGRP release, no change in glutamate release, reduced release of IL-1β but not TNFα	[54]
Rat CFA tibiotarsal joint	IA BoNT/A (dose-ranging) compared to CFA alone and saline control	All pain outcomes improved in a dose-dependent fashion. (Mechanical and thermal hyperalgesia) TRPV1 expression reduced but not transcription thought due to the observed reduced movement of TRPV1 to the cell membrane	[53]
Rat CFA ankle arthritis Plantar injection of capsaicin and formalin and plantar incision as standardized pain models. SNI model also included	BiTox – unique nonparalyzing botulinum toxin molecule	CFA induced reduced swelling, mechanical hyperalgesia but not reduced thermal hyperalgesia. No effect on acute pain from capsaicin or formalin, but reduced secondary mechanical hyperalgesia after plantar capsaicin injection. Plantar incision pain response reduced after day 2. Reduced neuropathic pain in the SNI model	[59]

CFA—complete Freund’s adjuvant-induced arthritis, COL—collagenase-induced osteoarthritis, BoNT/A—onabotulinumtoxinA, BoNT/B—rimabotulinumtoxinB, EMG — electromyography, BSA—bovine serum albumin, TMJ—temporal mandibular joint, SNI—spared nerve injury, SP—substance P, PGE2—prostaglandin E2.
